# Characteristics of complaints resulting in disciplinary actions against Danish GPs

**DOI:** 10.3109/02813432.2013.823768

**Published:** 2013-09

**Authors:** Søren Birkeland, Rene Depont Christensen, Niels Damsbo, Jakob Kragstrup

**Affiliations:** ^1^Research Unit of General Practice, Institute of Public Health, University of Southern Denmark, Odense, Denmark; ^2^Department and Research Unit of General Practice, Institute of Public Health, University of Copenhagen, Denmark

**Keywords:** Communication, Denmark, general practice, ICPC-2, jurisprudence, patient complaints

## Abstract

**Objective:**

The risk of being disciplined in connection with a complaint case causes distress to most general practitioners. The present study examined the characteristics of complaint cases resulting in disciplinary action.

**Material and methods:**

The Danish Patients’ Complaints Board's decisions concerning general practice in 2007 were examined. Information on the motives for complaining, as well as patient and general practitioner characteristics, was extracted and the association with case outcome (disciplinary or no disciplinary action) was analysed. Variables included complaint motives, patient gender and age, urgency of illness, cancer diagnosis, healthcare settings (daytime or out-of-hours services), and general practitioner gender and professional seniority.

**Results:**

Cases where the complaint motives involved a wish for placement of responsibility (OR = 2.35, *p* = 0.01) or a wish for a review of the general practitioner's competence (OR = 1.95, *p* = 0.02) were associated with increased odds of the general practitioner being disciplined. The odds of discipline decreased when the complaint was motivated by a feeling of being devalued (OR = 0.39, *p* = 0.02) or a request for an explanation (OR = 0.46, *p* = 0.01). With regard to patient and general practitioner characteristics, higher general practitioner professional seniority was associated with increased odds of discipline (OR = 1.97 per 20 additional years of professional seniority, *p* = 0.01). None of the other characteristics was statistically significantly associated with discipline in the multiple logistic regression model.

**Conclusion:**

Complaint motives and professional seniority were associated with decision outcomes. Further research is needed on the impact of professional seniority on performance.

Sanctions may be a major concern for general practitioners involved in a patient complaint case, but little is known about the characteristics of cases leading to criticism.

- Complaint cases motivated by a wish for placement of responsibility or a wish for review of the general practitioner's competence were associated with increased odds of the general practitioner being disciplined.
- When the patient's feeling of being devalued or a request for an explanation motivated the complaint, odds of discipline decreased.
- High professional seniority of the general practitioner was associated with increased odds of discipline.

## Introduction

The risk of receiving a patient complaint case seriously impacts on the work of medical doctors [1]. Professional self-esteem comes into play and especially in general practice the continuous patient–doctor relationship is at stake. For those general practitioners (GPs) receiving a complaint, the risk of being disciplined becomes a major concern. The characteristics of the complaints most likely to result in disciplinary action have received limited attention in the research literature. A Norwegian study suggested that male GPs and male patients are associated with complaint cases resulting in discipline. In that small study, 55 of the 108 cases (51%) concerned the out-of-hours service [2]. No larger studies exist, but it seems reasonable that apart from patient factors, communication issues [3], the motives for complaining (e.g. wish for punitive measures to be imposed, feelings of devaluation and humiliation, need for explanation, wish for particular health care persons to be held responsible), and the healthcare settings (daytime care or out-of-hours services) may potentially influence the outcome. Likewise, the kind of illness concerned (e.g. urgent vs. non-urgent) and health problem (e.g. cancer disease or not) may be issues of importance. Hence, a previous Norwegian study demonstrated that among the most serious complaints cases against general practitioners, the majority concerned urgent health care needs [4], and likewise cancer (and related patient deaths) has been suggested to play an important role in malpractice litigations [5,6].

This study aimed to analyse what characteristics (complaint motives, patients and GPs) were associated with being disciplined in connection with complaint cases against GPs.

## Material and methods

### Setting: Primary health care in Denmark and the disciplinary system

In 2006, more than 99% of the Danish population was listed with one of 3765 GPs working in approximately 2200 single-handed or partnership practices. Danish general practice is based on a contract with the tax-financed Danish National Health Insurance. The GPs act as gatekeepers with regard to the secondary healthcare system. Patients choose their GP and it is possible to change GP according to preferences (for a small fee). GPs are responsible for the care at all hours and GPs in a region collaborate in an organized out-of-hours service.

Patients who are dissatisfied with their GP may decide to file a written complaint. As in other countries, a national disciplinary system has been established to handle complaints about authorized healthcare professionals, including GPs. The board makes judgements only about professional conduct. Complaints not disputing professional conduct but only expressing dissatisfaction with the level of service (e.g. long waiting time or bad manners) are directed to the healthcare providers. Any compensation claims included in a complaint are handled in a separate Patient Insurance, while the Complaints Board makes decision about professional conduct. Alternatively, patients may file a complaint with the ‘Patientombuddet’ system, which handles complaints about courses of health care without intending named health professionals to be disciplined. Comparable complaints-handling authorities have been established in other countries [2,7]. At the initial stage, the board's secretariat clarifies the issues of the complaint. In this process the complainant and the defendant health professional will be heard. Like in other countries, the decision may be based on evaluations made by appointed experts. The final decision, however, is made by a five-person committee consisting of two public representatives, two representatives of the health profession concerned (e.g. GP specialists), and a chairperson who is a judge. The board has the authority to impose sanctions in the form of a discipline, the most commonly used being “criticism” or – until 1 January 2011 – disputing professional conduct (a milder comment). The other possible sanctions are “discipline with injunction”, or bringing the health professional before the prosecuting authority.

### Methods

All complaint case decisions concerning GPs completed in 2007 were reviewed. Information was extracted from case files (including the letter of complaint and all documents gathered in the handling process) and registered in a structured database.

The evaluation of motives behind the complaint was based on a review of the complaint letter. Based on the model described by Bismark and colleagues [8,9], the complainant motives were categorized in accordance with the patients’ expressed wish for: *explanation, placement of responsibility; quality improvement for future patients, review of the GP's competence*; *economic compensation, better level of general service*; *professional discipline*; and *other sanction*. A complaint may have more than one motive [8,9] and motives were, therefore, treated as eight separate variables in the statistical analysis. According to Bismark et al., the above eight motives cover the following four categories: Communication, correction, restoration, and sanction (see Table I). Additionally, it was noted whether the complaint was due to *feeling devalued* by the GP. Information was gathered on *patient gender, patient age*, and the illness concerned. With regard to patient illnesses, ICPC-2 coding was used. A *cancer* variable was constructed. Also, based on the ICPC-2 codes, a *serious urgent illness* variable was constructed after consensus between the authors (SB, JK, and ND). Deciding what illnesses to categorize as a *serious urgent illness* might imply difficulties. We chose only to consider diagnoses commonly resulting in death if untreated as *serious urgent illness*. Additionally, it was registered if the patient concerned, according to case management documentation, had died (*death of patient*). Other independent variables considered to be possible factors associated with discipline or potential confounders were healthcare settings (*daytime care* or *out-of-hours*), *general practitioner gender*, and *professional seniority* (years from graduation until event concerned). Information on GP *professional seniority* was gathered through manual look-up in a publicly available list covering Danish medical doctors [10].

**Table I.** Characteristics in complaint decisions relating to general practitioners (*n* = 571).

	n	%
Disciplined		
No	445	78
Yes	126	22
Complaint motives		
Communication	481	84
Explanation	300	53
Placement of responsibility	458	80
Correction	344	60
Quality improvement for future patients	214	37
Review of the GP's competence	328	57
Restoration	162	28
Economic compensation	124	22
Better level of general service	113	20
Sanction	106	19
Professional disciplinary action	95	17
Other sanction	96	17
Feeling devalued		
No	491	86
Yes	80	14
Patient characteristics		
Patient gender		
Female	335	59
Male	236	41
Cancer		
No	523	92
Yes	48	8
Serious urgent illness (see text)		
No	479	84
Yes	92	16
Death of patient		
No	507	89
Yes	64	11
General practitioner characteristics		
Healthcare settings		
Daytime care	335	59
Out-of-hours	236	41
General practitioner gender		
Female	170	30
Male	401	70

Finally, information on the decision outcome (discipline or no discipline) was noted. To analyse characteristics associated with discipline (the dependent variable), odds ratios (ORs) were estimated by means of a multiple logistic regression model including all the other mentioned variables. In some complaint cases decisions on professional conduct were made about two or more GPs. The unit used for the statistical analysis was the decision about individual GPs. All analyses were performed using STATA^®^, release 11.1 (StataCorp, College Station, TX, USA). P-values < 0.05 were considered statistically significant.

## Results

In 2007, the Danish Patient Complaints Board completed 571 decisions against individual GPs. Sample characteristics are given in Table I.

In 22% of decisions, the GP was disciplined. Criticism was expressed in 96 decisions (17%) and the professional conduct was disputed in 30 decisions (5%). The 96 decisions resulting in the GP being criticized included eight GPs being disciplined with injunction. One of these GPs was brought before the prosecuting authority, but the charge was later dropped. The average *patient age* was 45.3 years (range 0–91 years) and the average *professional seniority* of GPs was 22.2 years (range 0–47 years). The motives for complaining most often involved the categories of *Communication* and *Correction*, whilst the *Sanction* motive was encountered much less often. Table II presents the analysis of variables predictive of discipline. One case was omitted from the analysis because patient age was unknown.

**Table II.** Complaint case characteristics associated with receiving disciplinary action (n = 570).

	Odds ratio	p	95% CI	
Complaint motives				
Communication				
Explanation	0.46	0.01	0.26	0.80
Placement of responsibility	2.35	0.01	1.20	4.59
Correction				
Quality improvement for future patients	1.34	0.36	0.72	2.50
Review of the GP's competence	1.95	0.02	1.14	3.35
Restoration				
Economic compensation	1.45	0.26	0.76	2.75
Better level of general service	1.22	0.60	0.58	2.54
Sanction				
Professional disciplinary action	0.60	0.43	0.17	2.14
Other sanction	0.69	0.57	0.20	2.43
Feeling devalued				
No	1			
Yes	0.39	0.02	0.18	0.85
Patient characteristics				
Patient gender				
Female	1			
Male	0.91	0.66	0.59	1.40
Patient age (per year)	1.00	0.35	0.99	1.01
Cancer				
No	1			
Yes	0.86	0.73	0.37	2.00
Serious urgent illness				
No	1			
Yes	1.46	0.20	0.82	2.59
Death of patient				
No	1			
Yes	0.69	0.33	0.33	1.45
GP characteristics				
Healthcare settings				
Daytime care	1			
Out-of-hours	0.78	0.32	0.48	1.27
General practitioner gender				
Female	1			
Male	1.06	0.82	0.65	1.73
Professional seniority (per additional 20 years)	1.97	0.01	1.19	3.26

When including variables concerning complaint motives, *patient gender* and *patient age*, patient *serious urgent illness, cancer, death of patient*, healthcare settings (*daytime care* or *out-of-hours*), and *general practitioner gender* and *professional seniority* in a multiple logistic regression model, odds of discipline were halved when the complaint was motivated by *feeling devalued* (OR = 0.39, *p* = 0.02) or a request for an *explanation* (OR = 0.46, *p* = 0.01). However, when complaints involved a wish for *placement of responsibility* (OR = 2.35, *p* = 0.01) or a request for a *review of the GP's competence* (OR = 1.95, *p = *0.02) the odds of discipline doubled, just as with *professional seniority*: a GP with 20 years more seniority had doubled odds of being disciplined in connection with a complaint case (OR = 1.97, *p* = 0.01).

## Discussion

The key findings of this study are decreased odds of being disciplined when the complaint was motivated by *feeling devalued* or a request for an *explanation*. Increased odds of discipline were observed in complaint cases where the complaint was based on a wish for *placement of responsibility* or a wish for a *review of the GP's competence*. In addition, higher *professional seniority* was associated with increased odds of discipline. No statistical significance of *general practitioner gender* could be demonstrated.

The present study represents all complaint cases concerning GPs in Denmark completed during one year and is based on reliable register data and case files. The motives for complaining were recorded through review of the complaint letters and categorized according to the instrument produced by Bismark and colleagues [8]. The instrument has been validated previously [9]; among independent reviewers, the coding of 157 complaint letters matched in 83% (131) of cases. In the present study, no measure of inter-rater reliability was calculated even though, based on independent review and rating of a small test sample of complaint letters by two raters (authors SB and JK), it was concluded that the instrument could be transferred to the Danish complaint material. Nevertheless, when interpreting study results, it should be noted that any misclassification is likely to reduce the estimated associations between motives and decision outcomes.

As in the study from New Zealand by Bismark et al. [9], most complaints were based on the *Communication* and *Correction* motives. Bismark et al. did not investigate the impact of complaint motives on decision outcome, and we believe that our study is the first to do that. Feeling deserted or humiliated (in the sense of feeling objectified, insulted, ignored, or ridiculed) has been suggested to be important when patients lose trust in the doctor–patient relationship [11]. Accordingly, Beckman et al. [12] identified *Devaluing patient and/or family views* as the most important issue in almost one-third of malpractice suits and poor communication skills have previously been suggested to be predictive of receiving patient complaints [3]. In such situations our study suggests a decreased likelihood of being disciplined, perhaps because those feeling devalued or seeking punitive measures are overwhelmed by resentment whilst complaints motivated by a wish for *expression of responsibility* or a *review of the GP's competence* might mirror a relatively higher degree of matter-of-factness.

Two previous case-control studies (including hospital doctors) have demonstrated an association between increased professional seniority and complaint case discipline [13,14], but the findings were contradicted in a third study demonstrating decreased odds [15]. The suggested association between *professional seniority* and discipline may reflect seniority-dependent job contents: senior GPs might be those handling the most complex patient encounters. Anyhow, the study findings could not verify that *patient gender, patient age*, and *serious urgent illness* had any impact. Alternatively, the significance of *professional seniority* might reflect an unspecific *burnout* phenomenon. A comprehensive European cross-sectional questionnaire survey speaks in favour of this conception: this analysis of self-reports demonstrated a positive connection between professional seniority and burnout in terms of *emotional exhaustion* [16]. Hence, the question arises whether any association exists between complaint cases, GPs’ communication skills, and for example manifestations of burnout. Future studies should focus on the impact of GP professional seniority on performance.
